# Identification and molecular characterization of a psychrophilic GH1 β-glucosidase from the subtropical soil microorganism *Exiguobacterium* sp. GXG2

**DOI:** 10.1186/s13568-019-0873-7

**Published:** 2019-10-01

**Authors:** Bangqiao Yin, Hengsen Gu, Xueyan Mo, Yue Xu, Bing Yan, Quanwen Li, Qian Ou, Bo Wu, Chen Guo, Chengjian Jiang

**Affiliations:** 1 State Key Laboratory for Conservation and Utilization of Subtropical Agro-bioresources, College of Life Science and Technology, Guangxi University, 100 Daxue East Road, Nanning, 530004 Guangxi People’s Republic of China; 2 Guangxi Key Laboratory of Mangrove Conservation and Utilization, Guangxi Mangrove Research Center, Guangxi Academy of Sciences, 92 Changqing Road, Beihai, 536000 Guangxi People’s Republic of China; 3 Scientific Research Academy of Guangxi Environmental Protection, 5 Education Road, Nanning, 530022 Guangxi People’s Republic of China

**Keywords:** β-Glucosidase, Glycoside hydrolase family 1, *Exiguobacterium* sp., Subtropical soil microorganism

## Abstract

The products of bacterial β-glucosidases with favorable cold-adapted properties have industrial applications. A psychrophilic β-glucosidase gene named *bglG* from subtropical soil microorganism *Exiguobacterium* sp. GXG2 was isolated and characterized by function-based screening strategy. Results of multiple alignments showed that the derived protein BglG shared 45.7% identities with reviewed β-glucosidases in the UniProtKB/Swiss-Prot database. Functional characterization of the β-glucosidase BglG indicated that BglG was a 468 aa protein with a molecular weight of 53.2 kDa. The BglG showed the highest activity in pH 7.0 at 35 °C and exhibited consistently high levels of activity within low temperatures ranging from 5 to 35 °C. The BglG appeared to be a psychrophilic enzyme. The values of *K*_m_, *V*_max_, *k*_cat_, and *k*_cat_*/K*_m_ of recombinant BglG toward *ρ*NPG were 1.1 mM, 1.4 µg/mL/min, 12.7 s^−1^, and 11.5 mM/s, respectively. The specific enzyme activity of BglG was 12.14 U/mg. The metal ion of Ca^2+^ and Fe^3+^ could stimulate the activity of BglG, whereas Mn^2+^ inhibited the activity. The cold-adapted β-glucosidase BglG displayed remarkable biochemical properties, making it a potential candidate for future industrial applications.

## Introduction

β-1,4-Glucosidase, a pivotal rate-limiting enzyme of complex cellulose, breaks the β-1,4-glycosidic bond and generates diverse oligosaccharides, disaccharides, and alkyl β-d-glucosides (Volkov et al. 2014). A total of 308,372 β-glucosidase protein sequences had been identified in the NCBI protein database as of September 20, 2018. Among these protein sequences, most of the β-glucosidases were came from bacteria (282,519) (Chan et al. 2016; Chen et al. 2017; Sun et al. 2018), followed by fungi (11,863) (Guo et al. 2015; Hernandez-Guzman et al. 2016). The β-glucosidases have the characteristics of cold adaptation (Crespim et al. 2016; Ueda et al. 2010), heat resistance (Leis et al. 2018), salt tolerance (Lee et al. 2015), glucose tolerance (Chamoli et al. 2016; Chan et al. 2016), and organic solvent tolerance (Thomas et al. 2018) in extreme environments. β-Glucosidase is an indispensable component in the process of cellulose conversion to glucose (Sorensen et al. 2013). Production of bioactive aglycones by β-glucosidases are useful in food, feed, textiles, detergents and pharmaceuticals industrial area, such as soybean isoflavone aglycones (Kim et al. 2011) and ginsenosides (Xie et al. 2015). β-Glucosidase also has potential applications in the synthesis of oligosaccharides, alkyl glycosides and glycoconjugates (Singhania et al. 2017).

Cold-adapted β-glucosidases are highly desired nowadays. Low-temperature production is an industry requirement in order to maintain the flavor of food in the industry. The low catalytic temperature simultaneously saves a considerable amount of energy for industrial applications (Bhatia et al. 2002). A cold-tolerant β-glucosidase from Antarctica soil displayed the highest activity in the range of 10–40 °C (Crespim et al. 2016). A β-glucosidase from *Alteromonas* sp. L82 lost enzyme activity after treatment at 40 °C (Sun et al. 2018). The half-life of a β-glucosidase from *Bacillus* was 24 h at 40 °C, and its optimal temperature was 40 °C (Wu et al. 2018). Structure Loop L3 of a β-glucosidase from *Micrococcus antarcticus* played an important role in the psychrophilic attribute (Miao et al. 2016). The products of bacterial β-glucosidases with favorable cold-adapted properties have industrial applications.

Most β-glucosidase enzymes from bacteria belong to GH1 and GH3 family, which are grouped in the carbohydrate-active enzyme database (http://www.cazy.org) (Henrissat 1991). These grouping are based on similar structural characteristics, especially those related to conserved catalytic residues and the mechanism of enzymatic catalysis (de Giuseppe et al. 2014). The GH1 family members all have an identical catalytic domain of a typical (β/α)_8_ barrel and retaining catalytic mechanisms (Akhtar et al. 1976). Two glutamates are conserved residues in the active center (Stepper et al. 2013).

Presently, a psychrophilic β-glucosidase *bglG* gene was successfully cloned from the whole genome of subtropical soil microorganism *Exiguobacterium* sp. GXG2 through library construction and functional-based screening strategy. The derived amino acid protein BglG shared low similarity to the reviewed β-glucosidases and belonged to GH1 β-glucosidases. The effect of temperature, pH, metal ions, and EDTA on BglG was also determined. Structure and bioinformatics analyses of BglG were performed to further explore the function of β-glucosidase BglG.

## Materials and methods

### Vectors, strains, reagents, and kit

pGEM-3Zf (+), pETBlue-2, *E. coli* DH5α, and *Escherichia coli* Tuner™ (DE3) pLacI were all purchased from Novagen (Darmstadt, Germany). Fast Digest *Pst*I and Fast Digest *Eco*RI, DNA ligase, Pfu DNA polymerase, and λDNA/*Hin*dIII DNA Ladder were all purchased from New England BioLabs (Ipswich, USA). Esculin hydrate, *ρ*NPG, and all chemical reagents were bought from Sigma-Aldrich, Inc. (Darmstadt, Germany). Nickel–nitrilotriacetic acid (Ni–NTA) was bought from QIAGEN (Dusseldorf, Germany). Biospin Gel Extraction Kit, Biospin Bacteria Genomic DNA Extraction Kit, and Biospin PCR Purification Kit were all purchased from BioFlux (Tokyo, Japan).

### Construction and screening of gene library from *Exiguobacterium* sp. GXG2

In a previous study, a strain *Exiguobacterium* sp. GXG2 was isolated from subtropical soil samples in Nanning, Guangxi, China (22º50′15″N, 108°17′12″E) and deposited at the China Center for Type Culture Collection with the accession number of CGMCC16605 (Huang et al. 2011). The strain *Exiguobacterium* sp. GXG2 was rod-shaped, yellow, round, had neat edges and a bulging surface, and opaque. The strain was a Gram-positive bacterium, and the best production time was 2 h. The strain was inoculated in Mandels salts solution with 1% peptone, 0.5% yeast extract and 0.5% NaCl at 37 °C. The fermentation broth of the strain was collected by centrifuged at 12,000×*g* for 10 min at 4 °C. A gene library was constructed from the genome of *Exiguobacterium* sp. GXG2. The whole genome was randomly digested with restriction enzymes *Pst*I and *Eco*RI. The DNA fragments were ligated to the plasmid pGEM-3Zf (+) and chemically transformed into *E. coli* DH5α. The fragments were spread on Luria–Bertani (LB) agar plate supplemented with 0.05% of ferric ammonium citrate (w/v), 100 μg/mL ampicillin and 0.2% esculin (w/v), IPTG, and X-gal at 37 °C. The colonies with black phenotype sequenced at Sangon Biotech Co., Ltd. (Shanghai, China) were considered to show β-glucosidase activity.

### DNA sequence analysis and homology modeling

The online website (http://www.ncbi.nlm.nih.gov/gorf/orfig.cgi) was used to predict gene Open Reading Frames (ORFs). The similarity search for amino acids from ORFs was performed by the Basic Local Alignment Search Tool in the UniProtKB/Swiss-Pro database (https://www.uniprot.org). The websites ESPript 3.0 (http://espript.ibcp.fr/ESPript/cgi-bin/ESPript.cgi) and CLUSTALW (https://www.genome.jp/tools-bin/clustalw) were used for multiple sequence alignment. MEGA 6.0 software was used to construct a phylogenetic tree. The SWISS-MODEL server (https://www.swissmodel.expasy.org) and MODELLER program (version 9.18) were utilized for homology modeling. Four templates (PDB ID: 3ahx, le4i, luyq and 1tr1) were used to build the 3D structure of BglG. NAMD (Phillips et al. 2005) and VMD (Humphrey et al. 1996) were used for molecular dynamics simulation. AutoDock 4.2.6 was employed for molecular docking.

### Overexpression and purification of the recombinant BglG

The plasmid pETBlue-2 and *E. coli* Tuner™(DE3) pLacI were used as expression vector and strain, respectively, and cultured in LB medium containing ampicillin (100 μg/mL). Primers (B0F) CCGGAATTCTATGAAAATGCCAAAAGATTT and (B0R) TAACTGCAGTAGTTCAGCAGCACGTGTCG were used to verify the recombinant strain. The optimal temperature, IPTG concentration, and induction time were 20 °C, 0.8 mM, and 6 h, respectively. The induced OD of cells reached 0.6 at 600 nm, and the cells were collected by centrifugation at 9000 rpm for 20 min. The His60 Ni Gravity Column Purification Kit (Takara, Japan) was used the His-tagged protein purification with some modifications. Pre-cooled 2 mL His60 Ni ×Tractor Buffer was added to 100 mg cell pellet for resuspension and cell breakage by ultrasonication at 4 °C (37% power, 5 min, work interval of 10 s). The clear supernatant was collected by centrifuged at 12,000 rpm at 4 °C for 30 min. Add the sample to the equilibrated the His60 Ni Gravity Column (1 mL) and allow His-tagged protein to bind for 1 h at 4 °C. 10 column volumes of Elution Buffer was used for eluting and 500 μL fractions was collected at 4 °C according to the user manual protocols of the Kit. The targeted protein was analysis by sodium dodecyl sulfate-polyacrylamide gel electrophoresis (SDS-PAGE) (Crowe et al. 1994).

### BglG western blot analysis

The 6× His-tagged protein was confirmed further by western blot analysis using anti-6× His tag antibody. 15 µg of protein sample was separated by 12% SDS-PAGE and transferred to a polyvinylidene fluoride membrane (Merck, Millipore Ltd, Darmstadt, Germany). The 6× His-tagged protein were detected by incubation with (1:2000) anti-6× His tag antibody (ab1187) (Abcam, Cambridge, UK). The electrophoresis instrument and film transfer instrument were purchased from Bio-Rad (Hercules, CA, USA).

### Effect of pH and temperature on BglG

The effect of pH on BglG activity was performed at 0.1 M buffer (pH 4.0–8.0, Na_2_HPO_4_–citric acid buffer; pH 8.6–10.6, glycine–NaOH buffer; and pH 10.9–11.0, Na_2_HPO_4_–NaOH buffer) at 37 °C for 20 min. For pH stability, BglG was placed in different pH levels (pH 4.0–11.0) at 4 °C for 12 h, and enzyme activity was measured after the substrate *ρ*NPG was added at 35 °C for 20 min. The effect of temperature on BglG activity was performed in a disodium hydrogen phosphate (Na_2_HPO_4_)–citrate buffer of pH 7.0 at 5–65 °C (the interval is 5 °C) for 20 min. For thermostability, BglG was placed in pH 7.0 buffer at 5–65 °C for 1 h, and β-glucosidase activity was measured after the substrate *ρ*NPG was added at 35 °C for 20 min.

### Effect of EDTA and metal ions on BglG

Equal volumes of different chloride metal ions (CaCl_2_, CuCl_2_, FeCl_3_, BaCl_2_, LiCl, AlCl_3_, NaCl, MnCl_2_, MgCl_2_, FeCl_2_, and ZnCl_2_) and different concentrations of ethylenediaminetetraacetic acid (EDTA; 10, 20, 30, 40, and 50 mM) were added to the reaction system at 35 °C and pH 7.0 for 20 min. No metal ions and EDTA were added as a control.

### Determination of enzyme activity

The enzymatic reaction solution comprised pre-warmed 10 μL of 20 mM *ρ*NPG, pre-warmed 180 μL of Na_2_HPO_4_–citric acid buffer, and 50 μL of the appropriately diluted enzyme at 35 °C and pH 7.0. After 20 min of reaction, 200 μL of 2 mol/L NaCO_3_ solution was added to terminate the reaction, and 200 μL of the reacted liquid was placed in a 96-well plate. The released amount of *ρ*NPG was observed at 410 nm in an Epoch microplate spectrophotometer (BioTek Instruments, Inc., Winooski, USA). One unit (U) of β-glucosidase activity was defined as the amount of enzyme that releases 1 μmol *ρ*NP per minute.

### Substrate specificity assays of enzyme

Specificity of the BglG was measured by incubating the purified protein in glycine–NaOH (pH 7.0) that contains 1 mM of aryl-glycosides and saccharides (Merck KGaA, Darmstadt, Germany) at 35 °C for 20 min. Under the standard assay condition, the released *ρ*NP was determined. Moreover, the enzyme activity on laminaribiose, cellobiose, barley (1,3;1,4) β-d-glucan and soluble starch were determined by measuring the amount of reduced sugars with the dinitrosalicylic acid method.

### Nucleotide sequence accession number

The accession number of the BglG nucleotide sequence was AKA66375.1 in the GenBank database.

## Results

### Cloning and analysis of the β-glucosidase gene from *Exiguobacterium* sp. GXG2

A bacterial gene library of *Exiguobacterium* sp. GXG2 containing 10,080 positive clones was constructed with restriction enzyme sites *Pst*I and *Eco*RI. The entire library capacity was approximately 30 Mb, and the inserted DNA size was 3–10 kb. A substrate activity screening strategy was employed to obtain a positive clone. This clone, namely pGEMB0, was isolated from the metagenome library with black circle after incubated at 37 °C for 24 h (Additional file 1: Fig. S1). Vector from this positive clone was extracted and sequenced. The ORF encoded this β-glucosidase gene was designated as *bglG*. The size of the *bglG* gene was 1407 bp (Additional file 1: Fig. S2), and it encoded a 468 aa protein with a molecular weight of 53.2 kD, an isoelectric point of 5.06, and an overall hydrophilicity value of − 0.367 whose G + C content was 50.25%.

**Fig. 1:**
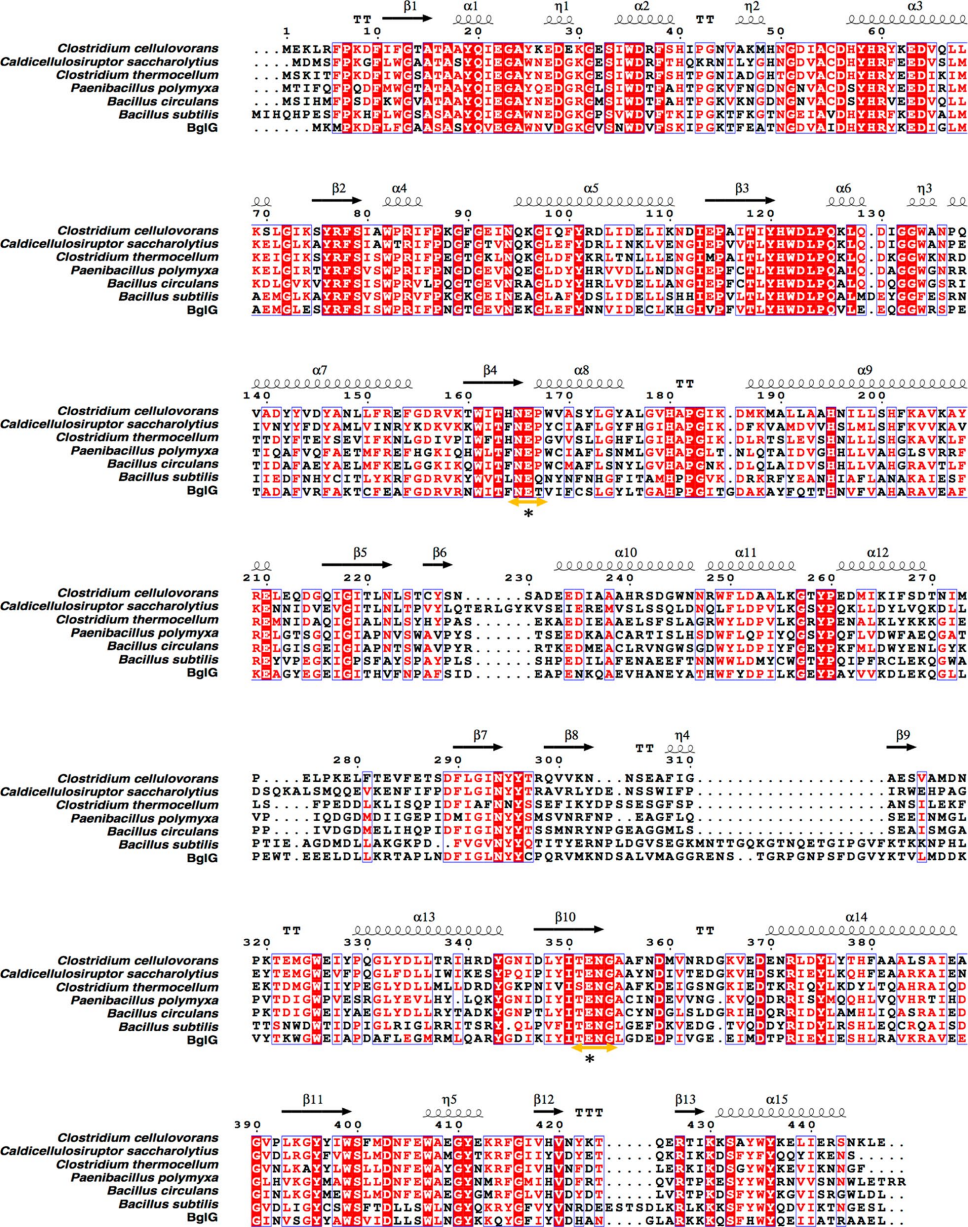
Multiple sequence alignment of BglG protein and other β-glucosidases. Sequence alignment was performed using the ClustalW and ESPript programs. Red box and white character indicated strict identity. Red character indicated similarity in a group. Blue frame indicated similarity across groups. α-Helices, β-strands, strict β-turns, and strict α-turns are displayed as coils, black arrows, TT, and TTT letters, respectively. Conserved β-glucosidase Asn-Glu-X (Pro/Gln/Thr) and X (Thr/Ser)-Glu-Asn-Gly) motifs are shown in the yellow double-direction arrows, and two conserved catalytic glutamate residues are depicted by asterisk. The sequences from top to bottom are *Clostridium cellulovorans* (PDB ID: 3ahx), *Caldicellulosiruptor saccharolyticus* (GenBank accession: CAA31087.1), *Clostridium thermocellum* (GenBank accession: GenBank accession: CAA42814.1), *Paenibacillus polymyxa* (GenBank accession: AAA22263.1), *Bacillus circulans* (GenBank accession: AAA22266.1), *Bacillus substilis* (GenBank accession: CAB12135.1), and BglG

**Fig. 2:**
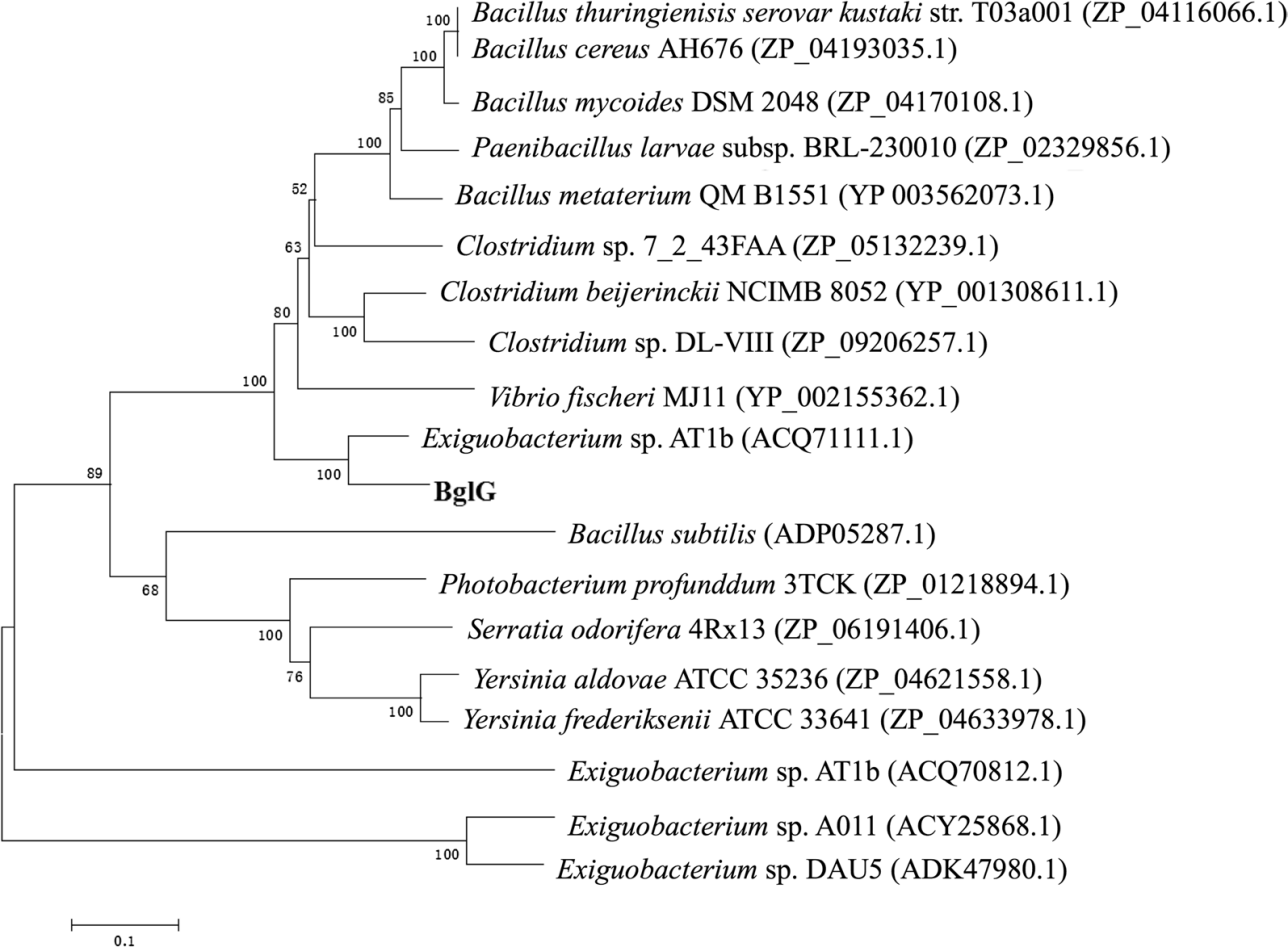
Phylogenetic tree analysis of BglG protein and other β-glucosidases. A phylogenetic tree was constructed using the neighbor-joining method with MEGA 6.0, and 1000 bootstrap replicates were indicated at branching points. BglG is shown in bold. The tree also shows the GenBank accession number and original genus of β-glucosidases

Multiple alignments of the BglG protein sequence with other GH1 β-glucosidases (Fig. 1) showed two highly conserved motifs of GH1 β-glucosidases named Asn-Glu-X (Pro/Gln/Thr) and X (Thr/Ser)-Glu-Asn-Gly, which were also found in BglG (Grabnitz et al. 1991). Two Glu amino acids were responsible for acid–base and nucleophilic catalysis in GH1 β-glucosidases (Moracci et al. 1995). A phylogenic tree (Fig. 2) using the neighbor-joining method was constructed by comparing BglG with some known β-glucosidase amino acid sequences with high similarity and consistency through the BLAST search program in the NCBI database (Fukumoto et al. 1988; Hayashimoto et al. 2005).

### Homology modeling and molecular dynamics of BglG

The BglG amino acid sequence was submitted to the SWISS-MODEL server using the Deep Viewer tool, and the template search was performed in the PDB database (Arnold et al. 2006; Phillips et al. 2005). The β-glucosidase template (PDB: 3ahx) from *Clostridium cellulovorans* and BglG shared the highest identity (40%) and similarity (59%) in the PDB database (Jeng et al. 2011). Multiple-template modeling was conducted by using four templates from *C. cellulovorans* (PDB: 3ahx, identity 40%, and similarity 59%) (Jeng et al. 2011), *Bacillus polymyxa* (PDB: 1e4i, identity 39%, and similarity 57%) (Sanz-Aparicio et al. 1998a), *Paenibacillus polymyxa* (PDB: 1uyq, identity 39%, and similarity 57%), and *B. polymyxa* (PDB: 1tr1, identity 39%, and similarity 57%) (Sanz-Aparicio et al. 1998b). Three output models were obtained by Python editing using the Modeller (version 9.18). Generally speaking, the first output model is the best one since its Molpdf value is the smallest and DOPE score is closer to 1 (Additional file 1: Table S1). PROCHECK and PROSA were used for assessing the generated structures (Addition file 1: Figs. S3 and S4). There are 91.1% amino acid were distributed in core area while only 0.2% in disallow area. The Z-score value of BglG is − 8.98, which located in the distribution range of the protein chain, indicating that the structure of BglG model is reasonable (Additional file 1: Table S2).

**Fig. 3:**
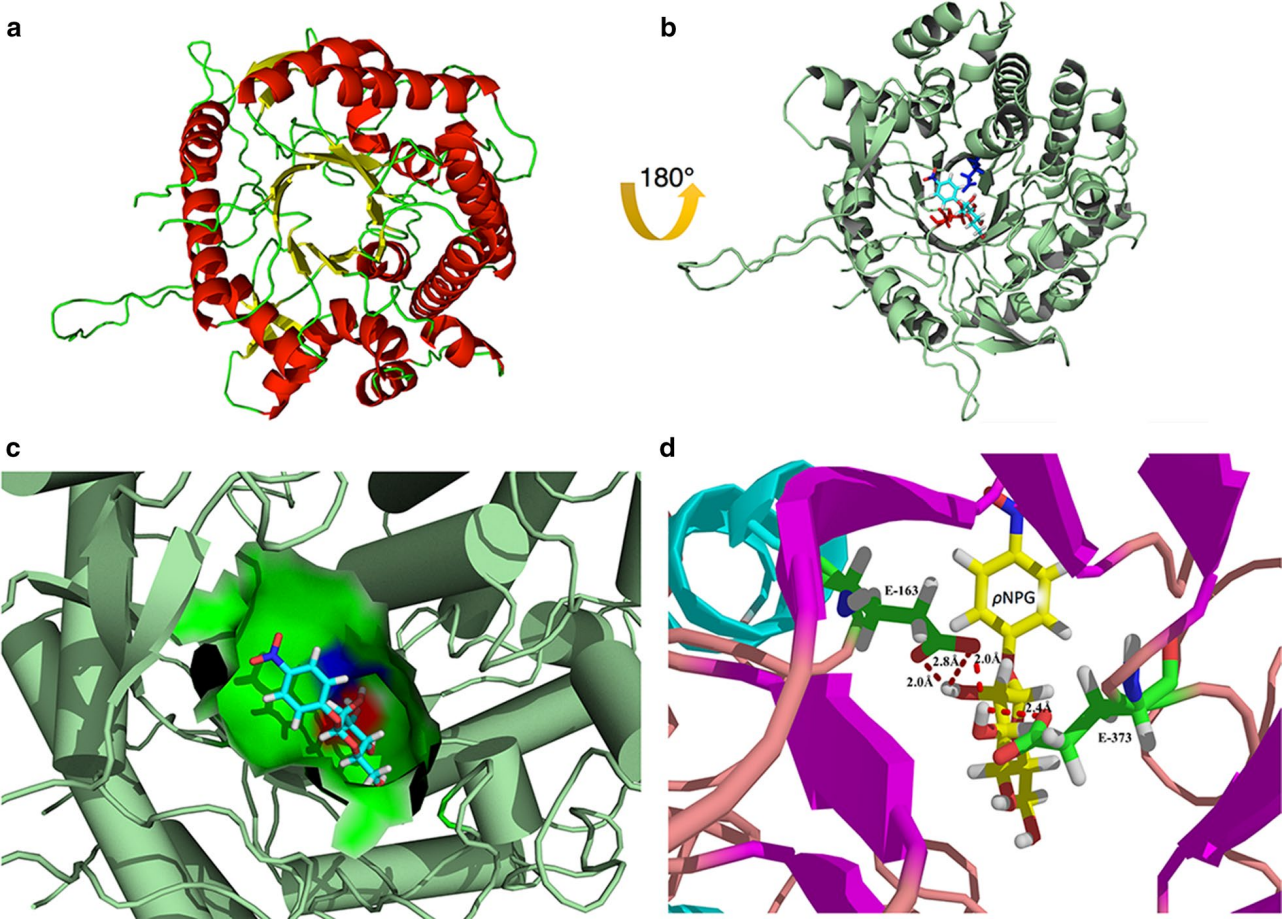
Homology modeling structure of BglG and docking models of BglG with *ρ*NPG. **a** The structure of (*β*/*α*)_8_-TIM barrel in the BglG model. α-Helix and β-strands are shown as red and yellow, respectively. **b** The cartoon representation of 3D structure of docking between the BglG model and *ρ*NPG. *ρ*NPG and catalytic residues (E163 and E373) are shown as cyan, red, and blue sticks, respectively. **c** The display of docking between the BglG model and *ρ*NPG. *ρ*NPG is shown as cyan sticks. E163 and E373 are shown in blue and red in the spit of the BglG surface, respectively. **d** Ball-and-stick representation of docking models of BglG with *ρ*NPG. E163 and E373 are shown as green sticks, and *ρ*NPG is shown as yellow sticks. Red dotted line represents possible hydrogen bonds

The BglG model contained a typical (β/α)_8_ barrel in overall structure, which exists in all β-glucosidases of the GH1 family (Fig. 3a) (Arthornthurasuk et al. 2018; Sun et al. 2018). An evident channel was formed on the center of the BglG enzyme, which is a non-polar hydrophobic environment also known as an enzyme reaction microenvironment (Wu et al. 2009).

Molecular docking of BglG was used as a receptor, whereas the substrate *ρ*NPG used as a ligand from the PubChem database was obtained through Autodock 4.2.6 software (Namasivayam and Gunther 2007). Figure 3b displays the cartoon structure of docking between BglG and *ρ*NPG, which demonstrated the structure binding sites located in the substrate and catalytic channel (Tribolo et al. 2007). In the pocket of BglG, the blue and red areas represented the amino acids Glu163 and Glu373, respectively (Fig. 3c). Five hydrogen bonds formed between *ρ*NPG and the surrounding amino acid residues in BglG (Fig. 3d). Three of these hydrogen bonds formed between E163 and the *ρ*NPG ligand, and the bond lengths were 2.0, 2.8 and 2.0 Å. However, E373 was 2.4 Å hydrogen bonded to *ρ*NPG. This phenomenon demonstrated that E163 and E373 were catalytic residues in the active center of BglG (Stepper et al. 2013).

### Expression and purification of BglG in *E. coli*

**Fig. 4:**
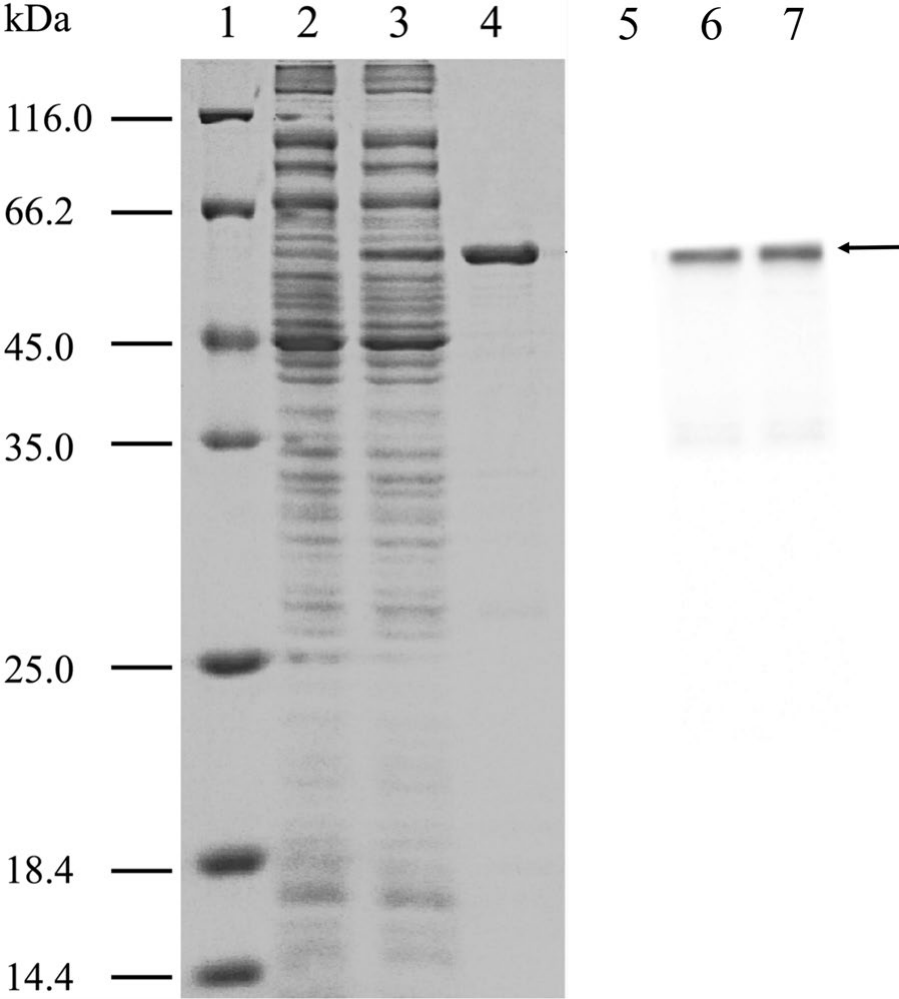
12% (w/v) SDS-PAGE and western blotting analysis of recombinant BglG. Lane 1, molecular mass standards. Lanes 2, total protein of Tuner(DE3)pLacI/pETBlue-2 (control). Lane 3, total protein of Tuner(DE3)pLacI harboring the recombinant *bglG* in pETBlue-2. Lane 4, purified recombinant BglG protein. Lane 5, Lane 6 and Land 7 shown western blotting analysis of expressed total protein of Tuner(DE3)pLacI/pETBlue-2 (control), total protein of Tuner(DE3)pLacI harboring the recombinant *bglG* in pETBlue-2 and purified recombinant BglG recognized by anti-his tag antibody, respectively. The black arrow indicates recombinant BglG. The black arrow indicates recombinant BglG

The *bglG* gene encoded the BglG protein and was subcloned using pETBlue-2 as an expression vector and Tuner (DE3) pLacI as an expression host. Crude cellulolytic lysate containing recombinant BglG was purified by the Ni–NTA column after being induced by 0.8 mM IPTG for 6 h at 20 °C and subjected to SDS-PAGE (Crowe et al. 1996). A considerable band at a size of 58.0 kDa, which was consistent with the aforementioned predicted size of BglG with a six-histidine tag sequence protein (Fig. 4), was observed. This protein was confirmed further by western blotting analysis using anti-6× His tag antibody. There was a single brand appeared in the position of the predicted size in the sample of purified BglG protein and the total protein extract with the recombinant vector (Fig. 4) when compared to the control.

### Physicochemical characterization of β-glucosidase BglG

#### pH and temperature

**Fig. 5:**
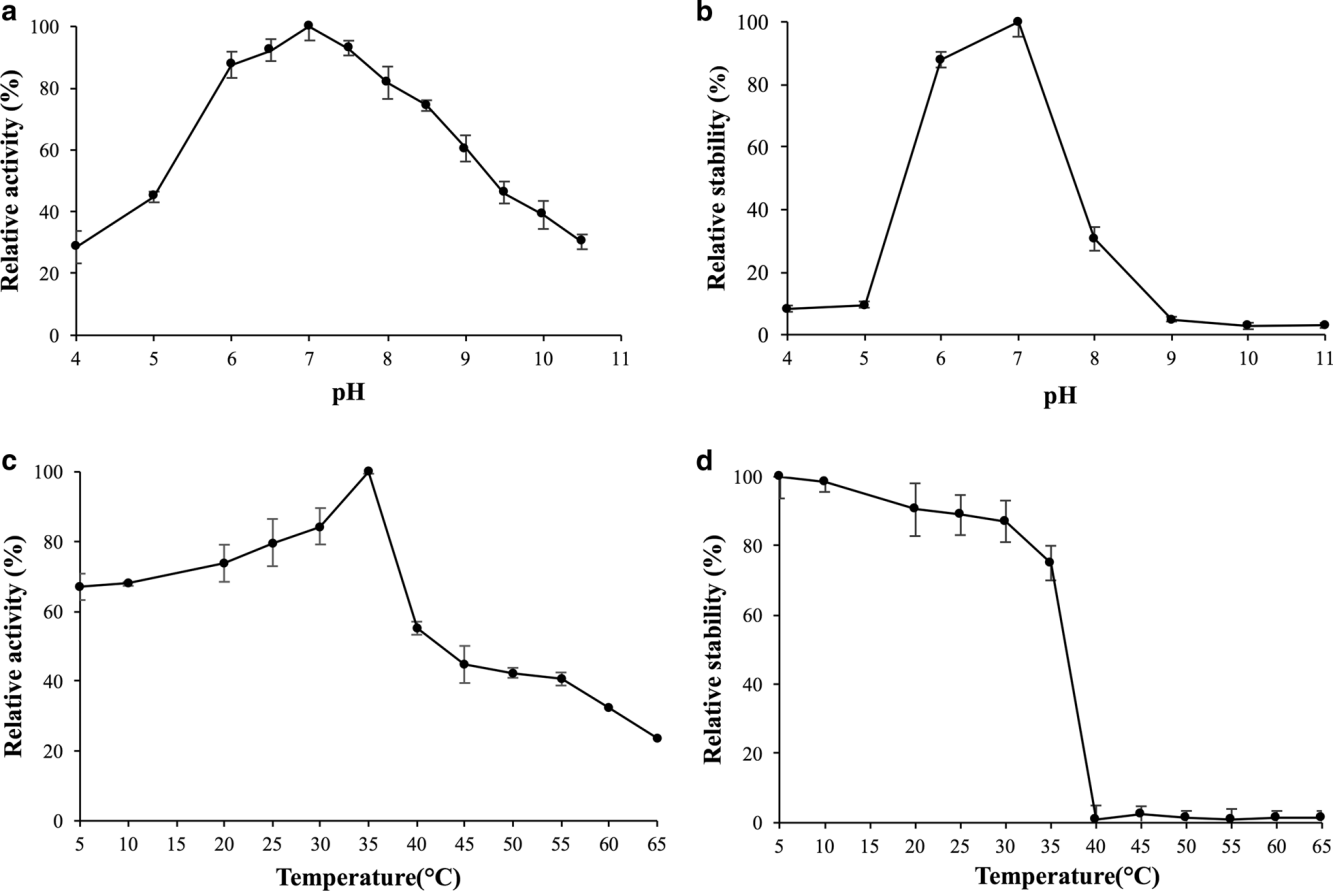
Enzymatic properties of recombinant BglG using *ρ*NPG as the substrate. **a** Effects of pH on enzyme activity. The enzyme activities were measured at 37 °C and pH of 4.0–11.0 in 0.1 M buffer. **b** Effect of pH on enzyme stability. The enzyme was mixed with 0.1 M buffers at pH 4.0–11.0 and incubated at 4 °C for 12 h. **c** Effects of temperature on enzyme activity. The enzyme activities were measured at 5–65 °C in pH 7.0. **d** Effects of temperature on enzyme stability. The enzyme was incubated at 5–65 °C for 1 h

Recombinant BglG exhibited high activity profiles at optimal pH of 7.0 (Fig. 5a). The pH stability curve for BglG protein showed that the enzymes were stable at pH 6.0–7.5 (Fig. 5b), retaining 70% enzyme activity. At optimal pH 7.0, recombinant BglG resulted in the optimal temperature of 35 °C (Fig. 5c). The temperature stability curve for the BglG protein showed that the enzymes were stable when the temperature was lower than 40 °C (Fig. 5d), which was similar to the β-glucosidases from *E. oxidotolerans* A011 (35 °C) (Shuilian Chen et al. 2010) and *Serratia* sp. TN49 (35 °C) (Zhou et al. 2011).

#### Metal ions and EDTA

Metal ions are often involved as an activator or inhibitor in the catalytic reaction of enzymes (Grasso et al. 2012; Temel and Kocyigit 2017). Therefore, the presence of an appropriate metal ion in the enzyme reaction system improved the catalytic efficiency of the enzymes. The effect of metal ions on BglG activity was determined for 20 min at pH 7.0 at 35 °C with no added metal ions as a control. Table 1 shows that most metal ions, such as Al^3+^, Fe^3+^, Ca^2+^, Mg^2+^, Li^+^, and Na^+^, exert a certain promoting effect on the enzyme activity of BglG. Among these metal ions, Ca^2+^ and Fe^3+^ strongly stimulated the enzyme activity of BglG, which demonstrated relative activities of 131% and 124%, respectively. However, Cu^2+^ and Mn^2+^ induced inhibiting effects on BglG activity. EDTA had a considerable inhibitory effect on the enzymatic activity of recombinant BglG. In the presence of 10 mM EDTA, the enzyme activity of BglG decreased by nearly 40%.

**Table 1:** Effects of metal ions and EDTA on the recombinant BglG activities

Compound	Concentration (mM)	Relative activity (%)
None	5	100.00 ± 0.60
Ca^2+^	5	131.12 ± 1.44
Cu^2+^	5	79.49 ± 2.30
Fe^3+^	5	124.83 ± 1.54
Ba^2+^	5	98.14 ± 0.81
Li^+^	5	112.59 ± 2.17
Al^3+^	5	120.40 ± 1.03
Na^+^	5	112.94 ± 2.81
Mn^2+^	5	72.84 ± 0.96
Mg^2+^	5	111.89 ± 3.40
Fe^2+^	5	94.06 ± 5.53
Zn^2+^	5	84.50 ± 4.12
EDTA	10	63.85 ± 1.25
EDTA	20	60.53 ± 0.29
EDTA	30	58.71 ± 0.50
EDTA	40	56.55 ± 0.76
EDTA	50	54.89 ± 0.76

#### Enzyme kinetic analysis and substrate specificity

The initial velocity of the reaction of the BglG protein was measured under different substrate concentrations (1–10 mM) at pH 7.0 and 35 °C. Lineweaver–Burk plots were used to determine reaction kinetic parameters of purified BglG (Fjellstedt and Schlesselman 1977). *K*_m_, *V*_max_, *k*_cat_, and *k*_cat_*/K*_m_ obtained for enzymes of recombinant BglG toward *ρ*NPG were 1.1 mM, 1.4 µg/mL/min, 12.7 s^−1^, and 11.5 mM/s, respectively (Table 2). The specific enzyme activity of BglG was 12.14 U/mg.

**Table 2:** Properties of β-glucosidase from various resources in the past 4 years

Sources	*K*_m_ (mM)	*V*_max_ (U/mg)	*k*_cat_ (s^−1^)	*k*_cat_*/K*_m_ (mM/s)	pH	Temperature (°C)	References
*Alteromonas* sp.	0.55	83.60	74.30	135.10	7.0	40	Sun et al. (2018)
*B. cellulosilyticus*	2.97	66.20	208.70	70.30	7.0	40	Wu et al. (2018)
Soil metagenome	0.49	10.81	2.90	5.90	7.0	37	Gomes-Pepe et al. (2016)
*C. crescentus*	0.24	0.04	0.06	0.27	6.0	50	Justo et al. (2015)
*T. maritima.*	0.56	238.40	187.10	333.9	5.0–7.0	80–100	Mehmood et al. (2014)
*Exiguobacterium* sp.	1.1	12.14	12.7	11.5	7.0	35	This work

The relative hydrolysis rates of some artificial and natural substrates by purified BglG were determinate (Table 3). BglG can hydrolyze *ρ*NPG effectively. The substrates *ρ*NP-β-d-cellobioside and cellobiose were hydrolyzed at 68% and 41% of rate for *ρ*NPG, respectively. It could not degrade the *ρ*NP-β-d-galactopyranoside, *ρ*NP-β-d-xylopyranoside, laminaribiose, barley (1,3;1,4)-β-d-glucan and souble starch.

**Table 3:** Substrate specificity of recombinant BglG

Substrate	Linkage of glycosyl group	Relative activity (%)
*ρ*NP-β-d-glucoside	βGlc	100^a^
*ρ*NP-β-d-galactopyranoside	βGal	ND
*ρ*NP-β-d-xylopyranoside	βXyl	ND
*ρ*NP-β-d-cellobioside	β(1,4)Glc	68
Laminaribiose	Glcβ(1,3)Glc	ND
Cellobiose	Glcβ(1,4)Glc	41
Barley (1,3;1,4)-β-d-glucan	β(1,3/1,4)Glc	ND
Soluble starch	αGlc	ND

*ND* not detected

100^a^: Activity on *ρ*NP-β-d-glucoside was defined as 100%, which corresponds to specific activity of 12.1 U/mg of BglG

## Discussion

BLAST analysis of the deduced BglG sequence in the UniProtKB/Swiss-Prot database with other reported β-glucosidases with manual annotation and experimental evidence displayed that BglG shared the highest identity (45.7%) with β-glucosidase BglC from *Bacillus subtilis* (GenBank: CAB12135.1) (Setlow et al. 2004). Blast results also showed that BglG shared approximately 39.7%, 39.1%, 40.3%, and 39.4% identities with some β-glucosidases in the UniProtKB/Swiss-Prot database. The four β-glucosidases were BglA from *C. thermocellum* (strain ATCC 27405) (GenBank: CAA42814.1) (Grabnitz et al. 1991) and β-glucosidases from *P. polymyxa* (GenBank: AAA22263.1) (Gonzalez-Candelas et al. 1990), *Bacillus circulans* (GenBank: AAA22266.1) (Paavilainen et al. 1993), and *Caldicellulosiruptor saccharolyticu*s (GenBank: CAA31087.1) (Love et al. 1988). These results showed that BglG had low similarity to the manually annotated and reviewed β-glucosidase protein sequences.

BglG shared a common ancestor with β-glucosidases derived from *Exiguobacterium* sp. (Vishnivetskaya et al. 2011), *Vibrio fischeri*, *Clostridium* sp., *Bacillu*s sp., and *Paenibacillus* sp. Moreover, BglG was the closest evolutionary relative to β-glucosidase from *Exiguobacterium* sp. AT1b. Multiple-template modeling was more accurate than single-template modeling. Ramachandran plots and PROSA evaluation of the multi-template generation BglG model showed that the predicted BglG model was reasonable in terms of structure and energy (Carrascoza et al. 2014; Webb and Sali 2016; Wiederstein and Sippl 2007).

Two Glus (E163 and E373) were highly conserved in the active center of the GH1 family BglG through NAMD and VMD software (Humphrey et al. 1996; Phillips et al. 2005), which are involved in acid–base and nucleophilic catalysis, respectively (Badieyan et al. 2012; Liu et al. 2011). When the E163 and E373 sites were simulated to mutate to Gly, the possible hydrogen bond formations were reduced, leading to changes in the spatial structure of BglG. This phenomenon indirectly proved that the two sites were the active sites of the enzyme (Additional file 1: Fig. S5).

The molecular masses of BglG were similar to those of β-glucosidases from *Exiguobacterium oxidotolerans* A011 (51.6 kDa) (Shuilian Chen et al. 2010), but different from β-glucosidases from *Trichoderma reesei* (76.0 kDa) (Chen et al. 2011).

The optimal pH of BglG corresponded to the β-glucosidase from *E. oxidotolerans* A011 (pH 7.0) (Shuilian Chen et al. 2010), whereas most of the β-glucosidases, such as β-glucosidases from *Aspergillus niger* (pH 5.0) (Wei et al. 2007), *T. harzianum* (pH 5.0) (Soo-In Yun et al. 2011), *Sporidiobolus pararoseus* yeast strain (pH 5.0) (Baffi et al. 2011), and *Thermoascus aurantiacus* (pH 4.5) (Parry et al. 2001), shared optimal pH at 5.0 or lower than 5.0. BglG retained over 80% activities after incubation at 5–35 °C for 1 h. However, β-glucosidase activity was rapidly lost after incubation at 40 °C for 1 h. Compared with other known β-glucosidases from *T. reesei* (70 °C) (Chen et al. 2011), *T*. *aurantiacus* (80 °C) (Parry et al. 2001), *A. niger* (66 °C) (Wei et al. 2007), *Fervidobacterium islandicum* (80–100 °C) (Jabbour et al. 2012), *T. harzianum* (45 °C) (Soo-In Yun et al. 2011), and *Sporidiobolus pararoseus* yeast strain (50 °C) (Baffi et al. 2011), BglG was clearly a psychrophilic β-glucosidase.

BglG is shown highly specificity toward β(1,4) linkages substrates. It could not break β-glucans containing β(1,3), β(1,4) and β(1,3/1,4) linkages. It also do not hydrolyzed βGal, βXyl and αGlc glycosidic linkages. The specific enzyme activity of BglG was 5- and 303-folds higher than those of β-glucosidases from almonds and *Caulobacter crescentus* (Hernandez-Maya and Canizares-Macias 2018; Justo et al. 2015). This result demonstrated that BglG had higher affinity to combine the substrate *ρ*NPG than a β-glucosidase from *B. cellulosilyticus* because *K*_m_ of β-glucosidase from *B. cellulosilyticus* was 2.3-fold higher than that of BglG. The turnover efficiency (*k*_cat_) of BglG was 4.3-fold higher than that of β-glucosidase from soil metagenome (Gomes-Pepe et al. 2016), whereas *k*_cat_ of BglG was 5.8-, 16.4-, and 14.7-folds lower than those of most reported β-glucosidases in the past 4 years from *Alteromonas* sp., *B. cellulosilyticus*, and *Thermotoga maritima* (Mehmood et al. 2014; Sun et al. 2018; Wu et al. 2018). Therefore, the psychrophilic β-glucosidase, BglG, needs modification through protein engineering in the future.

In this study, a β-glucosidase gene was firstly isolated and characterized by function-based screening strategy from subtropical soil microorganisms. The recombinant BglG was demonstrated as a member of the GH1 family and Glu163 and Glu373 are two key catalytic sites in the active center of the BglG according to the analysis results of physicochemical properties, key site prediction, homology modeling, and molecular docking. The detailed biochemical characterization of BglG had also been performed, and BglG had been found to show unexpectedly high activity across a low-temperature range (5–35 °C). This raw material also offered a possibility to improve the properties of BglG through protein engineering for further industrial demand.

## Supplementary information

**Additional file 1. Table S1.** The results of homology modeling of BglG by multiple-template. **Table S2.** The evaluation of and multi-template model of BglG. **Fig. S1.** Functional screening of gene library. **Fig. S2.** Agarose gel electrophoresis of *bglG* gene. **Fig. S3.** The ramachandran plot of BglG multi-template model. **Fig. S4.** The PROSA evaluation of BglG multi-template model. **Fig. S4.** The PROSA evaluation of BglG multi-template model. **Fig. S5.** Compare the changes in surrounding residues of active sites E163 and E373 after mutant to glycine.

## Data Availability

Not applicable.
