# Supplementary material for: Identification and molecular characterization of a psychrophilic GH1 β-glucosidase from the subtropical soil microorganism Exiguobacterium sp. GXG2
Source: AMB Express. 2019 Oct 1;9:159. doi: 10.1186/s13568-019-0873-7 (PMC6773797; doi:10.1186/s13568-019-0873-7)
Supplement: Supplementary file 1 — Additional file 1. Table S1. The results of homology modeling of BglG by multiple-template. Table S2. The evaluation of and multi-template model of BglG. Fig. S1. Functional screening of gene library. Fig. S2. Agarose gel electrophoresis of bglG gene. Fig. S3. The ramachandran plot of BglG multi-template model. Fig. S4. The PROSA evaluation of BglG multi-template model. Fig. S4. The PROSA evaluation of BglG multi-template model. Fig. S5. Compare the changes in surrounding residues of active sites E163 and E373 after mutant to glycine. [file 13568_2019_873_MOESM1_ESM.pdf]

Additional file 1. Additional tables and figures

Title: Identification and molecular characterization of a psychrophilic GH1 β-glucosidase from the subtropical soil microorganism *Exiguobacterium* sp. GXG2

Authors: Bangqiao Yin[1*], Hengsen Gu[1], Yue Xu[1], Bing Yan[2], Quanwen Li[1], Qian Ou[1], Bo Wu[1], Chen Guo[3**], and Chengjian Jiang[1, 2***]

Author details:

[1] State Key Laboratory for Conservation and Utilization of Subtropical Agro-bioresources, College of Life Science and Technology, Guangxi University, 100 Daxue East Road, Nanning, Guangxi, 530004, P.R. China.

[2] Guangxi Key Laboratory of Mangrove Conservation and Utilization, Guangxi Mangrove Research Center, Guangxi Academy of Sciences, 92 Changqing Road, Beihai, Guangxi, 536000, P.R. China.

[3] Scientific Research Academy of Guangxi Environmental Protection, 5 Education Road, Nanning, Guangxi 530022, P.R. China.

[*], [**]and [***]: Corresponding Author

[*]Bangqiao Yin:

Tel: +86-771-3239403; Fax: +86-771-3237873; E-mail: yinbangqiao@163.com

[**]Chen Guo:

Tel: +86-771-3239403; Fax: +86-771-3237873; E-mail: 394176889@qq.com

[**]Chengjian Jiang:

Tel: +86-771-3239403; Fax: +86-771-3237873; E-mail: jiangcj0520@gmail.com

Additional Tables

Table S1 The results of homology modeling of BglG by multiple-template

Name of Model	molpdf	DOPE score	GA341 score
query.B99990001.pdb	13346.14160	-58236.37891	1.00000
query.B99990002.pdb	13493.02832	-57447.93359	1.00000
query.B99990003.pdb	13395.54688	-57681.30078	1.00000

Table S2 The evaluation of and multi-template model of BglG

Model	Ramachandran plot				G-factor			Errat
	Core	Allow	Gener	Disall	Dihedrals	Covalent	Overall	
query.B99990001.pdb	91.1%	7.4%	1.2%	0.2%	0.00	-0.18	-0.07	64.396

Additional figure legends

Fig. S1 Functional screening of gene library. Hydrolyzing zones produced by *E.coli* strains harbored positive β-glucosidase genes on agar plates, containing ampicillin, esculin hydrate, and ferric ammonium citrate. A pGEMB0 clone was indicated by a black arrow.

Fig. S2 Agarose gel electrophoresis of *bglG* gene. Lane 1: 1kb DNA marker. Lane 2 and 3: *bglG* gene from pGEMB0.

Fig. S3 The ramachandran plot of BglG multi-template model.

Fig. S4 The PROSA evaluation of BglG multi-template model.

Fig. S5 Compare the changes in surrounding residues of active sites E163 and E373 after mutant to glycine. (A) The distribution of all amino acid residues 4 Å away from E163 site in BglG. Four residues (Thr218, Phe167, Ile166 and Asn295) were formed hydrogen bonds with Glu163 (bond length of 2.7, 3.1, 3.1 and 3.2 Å) among 11 residues. **(B)** The distribution of all amino acid residues 4 Å away from A163 site in BglG. After mutate E163 to A163, the number of amino acid residues about 4.0 Å from A163 was significantly reduced to 7 and only leave 3 hydrogen bonds. **(C)** The distribution of all amino acid residues 4 Å away from E373 site in BglG. Glu373 was hydrogen bonded to Asn374, Asn295, Asn163 and Leu294 with bond length of 2.6 and 3.0, 3.3 and 3.4 Å, respectively. **(D)** The distribution of all amino acid residues 4 Å away from A373 site in BglG. After mutate E373 to A373, A373 forms only two hydrogen bonds with Asn374 and Leu294 (3.0 and 3.4 Å). Red dotted line represents hydrogen bond. The active site residues are showed as red for the C_α atoms, while the residues around active sites are showed as green sticks.

Fig. S1

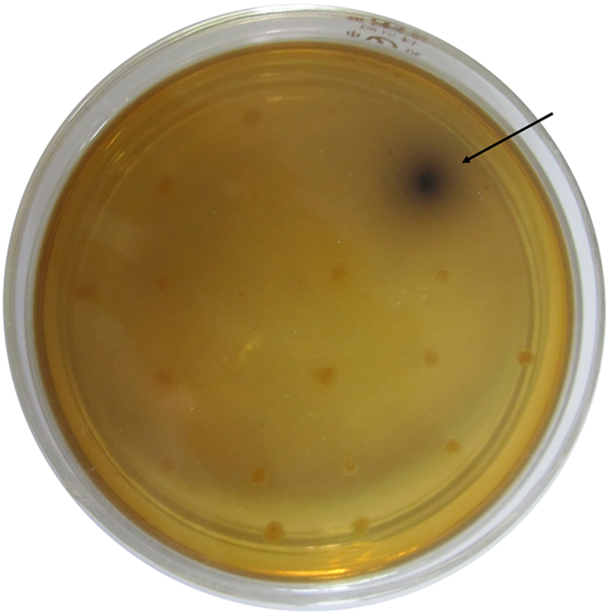

Fig. S2

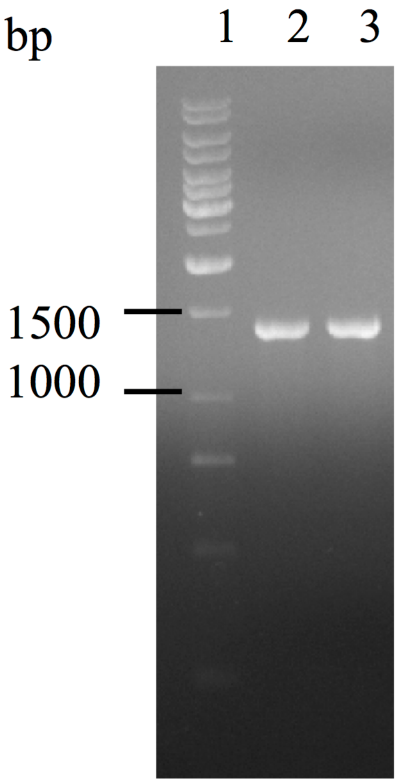

Fig. S3

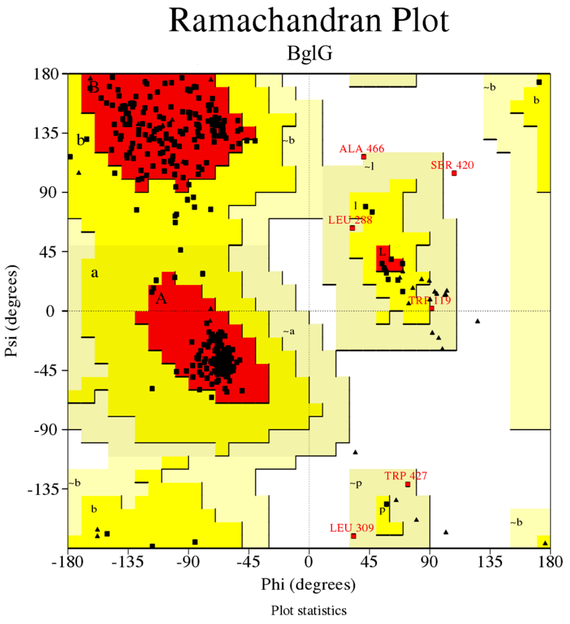
Ramachandran Plot
BglG
Psi (degrees)
Phi (degrees)
ALA 466
SER 420
LEU 288
TRP 119
TRP 427
LEU 309
Plot statistics

Fig. S4

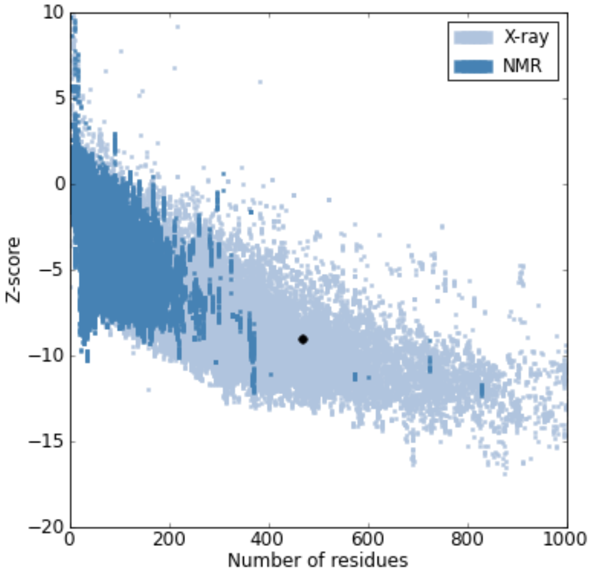
X-ray
NMR
Z-score
10
5
0
−5
−10
−15
−20
0
200
400
600
800
1000
Number of residues

Fig. S5

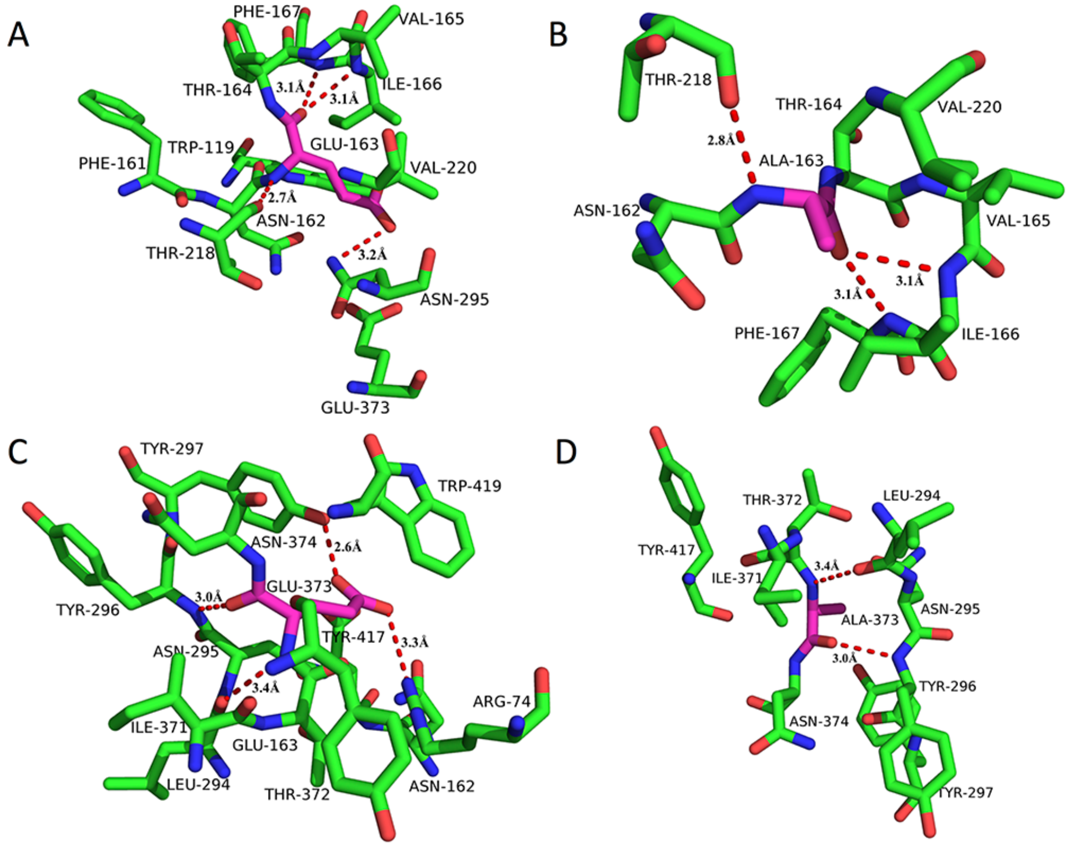
A
PHE-167
VAL-165
THR-164
3.1Å
3.1Å
ILE-166
TRP-119
GLU-163
PHE-161
VAL-220
2.7Å
ASN-162
THR-218
3.2Å
ASN-295
GLU-373
B
THR-218
THR-164
VAL-220
2.8Å
ALA-163
ASN-162
VAL-165
3.1Å
3.1Å
PHE-167
ILE-166
C
TYR-297
TRP-419
ASN-374
2.6Å
GLU-373
TYR-296
3.0Å
TYR-417
3.3Å
ASN-295
3.4Å
ARG-74
ILE-371
GLU-163
LEU-294
THR-372
ASN-162
D
THR-372
LEU-294
TYR-417
ILE-371
3.4Å
ASN-295
ALA-373
3.0Å
TYR-296
ASN-374
TYR-297
